# Genome Wide Association Study (GWAS) between Attention Deficit Hyperactivity Disorder (ADHD) and Obsessive Compulsive Disorder (OCD)

**DOI:** 10.3389/fnmol.2017.00083

**Published:** 2017-03-23

**Authors:** McKenzie L. Ritter, Wei Guo, Jack F. Samuels, Ying Wang, Paul S. Nestadt, Janice Krasnow, Benjamin D. Greenberg, Abby J. Fyer, James T. McCracken, Daniel A. Geller, Dennis L. Murphy, James A. Knowles, Marco A. Grados, Mark A. Riddle, Steven A. Rasmussen, Nicole C. McLaughlin, Erika L. Nurmi, Kathleen D. Askland, Bernadette Cullen, John Piacentini, David L. Pauls, Joseph Bienvenu, Evelyn Stewart, Fernando S. Goes, Brion Maher, Ann E. Pulver, Manuel Mattheisen, Ji Qian, Gerald Nestadt, Yin Yao Shugart

**Affiliations:** ^1^Unit on Statistical Genomics, National Institute of Mental Health, National Institutes of Health (NIH)Bethesda, MD, USA; ^2^Department of Psychiatry and Behavioral Sciences, Johns Hopkins University School of MedicineBaltimore, MD, USA; ^3^Department of Mental Health, Johns Hopkins University Bloomberg School of Public HealthBaltimore, MD, USA; ^4^Department of Psychiatry, Faculty of Medicine, University of TorontoToronto, ON, Canada; ^5^New York State Psychiatric Institute, College of Physicians and Surgeons at Columbia UniversityNew York, NY, USA; ^6^Department of Psychiatry and Biobehavioral Sciences, University of California, Los Angeles School of MedicineLos Angeles, CA, USA; ^7^Department of Psychiatry, Massachusetts General Hospital and Harvard Medical SchoolBoston, MA, USA; ^8^Laboratory of Clinical Science, National Institute of Mental HealthBethesda, MD, USA; ^9^Department of Psychiatry and Behavioral Sciences, Keck School of Medicine at the University of Southern CaliforniaLos Angeles, CA, USA; ^10^Department of Psychiatry, University of British ColumbiaVancouver, BC, Canada; ^11^Department of Biomedicine and Center for Integrated Sequencing (iSEQ), Aarhus UniversityAarhus, Denmark; ^12^Department of Biostatistics, Harvard School of Public HealthBoston, MA, USA; ^13^Department of Genomic Mathematics, University of BonnBonn, Germany; ^14^State Key Laboratory of Genetic Engineering, Life Science Institutes, Fudan UniversityShanghai, China

**Keywords:** ADHD, OCD, GWAS, meta-analysis, polygenic score, protein-protein link analysis, eQTL

## Abstract

**Objective:** The aim of this study was to identify any potential genetic overlap between attention deficit hyperactivity disorder (ADHD) and obsessive compulsive disorder (OCD). We hypothesized that since these disorders share a sub-phenotype, they may share common risk alleles. In this manuscript, we report the overlap found between these two disorders.

**Methods:** A meta-analysis was conducted between ADHD and OCD, and polygenic risk scores (PRS) were calculated for both disorders. In addition, a protein-protein analysis was completed in order to examine the interactions between proteins; *p*-values for the protein-protein interaction analysis was calculated using permutation.

**Conclusion:** None of the single nucleotide polymorphisms (SNPs) reached genome wide significance and there was little evidence of genetic overlap between ADHD and OCD.

## Introduction

Attention deficit hyperactivity disorder (ADHD) and obsessive compulsive disorder (OCD) are two neurodevelopmental disorders with their onset in childhood. They are two of the most common psychiatric disorders found in pediatric populations. Approximately 5% of the population worldwide has ADHD (Simon et al., 2009). ADHD is most often characterized by inattention, hyperactivity and impulsivity and affects twice as many males compared to females (Polanczyk et al., 2007). OCD is characterized by recurring obsessions and/or compulsions. Obsessions are unwanted thoughts, ideas and impulses that occur more than once, while compulsions are repetitive behaviors that are driven by the obsessions (American Psychiatric Association, 2016). OCD affects up to 2% of the world’s population and is 4.5 times more common in males than females (Eaton et al., 2008).

The comorbidity of ADHD and OCD was found to range vastly from 10% to 50% (Geller et al., 1996; Masi et al., 2006; Brem et al., 2014; Abramovitch et al., 2015). Some studies may have exhibited high comorbidity rates because ADHD often presents with inattention and distractibility, which could be misdiagnosed as OCD (Geller et al., 2002). This shared clinical feature of the disorders often makes diagnosis difficult. Geller et al. (2002) indicated that the ADHD symptoms found in OCD patients are not sub-symptoms of OCD, but instead true comorbidity of the disorders.

Several studies have been conducted that to examine the overlapping sub-phenotypes between ADHD and OCD. Sheppard et al. (2010) documented that ADHD and OCD share symptoms of inattention and distraction and co-segregation in families. Recently, Park et al. (2016) pointed out that hoarding; a common symptom observed in OCD, may be linked to executive functioning deficits were found to be associated with ADHD (Park et al., 2016). Additionally, individuals with ADHD and OCD are thought to share diminished inhibitory control, which is conveyed through impulsivity in ADHD, and poor control of obsessions and compulsions in OCD (Norman et al., 2016). The recent evidence of these overlapping symptoms between ADHD and OCD serve as the motivation for this study. Because the clinical relationship has been well established in the literature (Geller et al., 1996; Masi et al., 2006), we sought to examine the genetic relationship between ADHD and OCD.

There have been studies for assessing potential genetic overlap between two neurological disorders using genome wide association study (GWAS) data. Davis et al. (2013) conducted an analysis between Tourette’s Syndrome (TS) and OCD, utilizing a similar approach to our study. Davis et al. (2013) found overlap between the two disorders with a genetic correlation of 0.41 (*p*-value < 0.002). This also justified our efforts to conduct an analysis between ADHD and OCD, which to our knowledge had not been previously studied, as the Davis et al. (2013) study used statistical tests that yielded significant results for a genetic link between TS and OCD.

To achieve our research goal, we performed a meta-analysis between the ADHD (*N* = 3351) and OCD (*N* = 5415) samples. The ADHD sample contained 2064 trios, 896 cases, and 2455 controls. The OCD sample consisted of 2998 individuals from nuclear families. Further, the meta-analysis results are examined for the enrichment of functional single nucleotide polymorphisms (SNPs) that were previously associated with gene expression levels. Additionally, polygenic risk score (PRS) analyses were completed to test the hypothesis that multiple genes of small effect jointly contribute to the susceptibility of ADHD and OCD. PRS analyses were also conducted to investigate the genetic relationship between these two disorders. Furthermore, an additional analysis was conducted to examine protein-protein interactions to examine potential common pathways between proteins. Lastly, an expression quantitative trait locus (eQTL) analysis was carried out to identify non-randomly occurring genes that are associated with the prefrontal cortex region. We used the nominated genes generated by two additional approaches: eQTL and Disease Association Protein-Protein Link Evaluator (DAPPLE)^1^, to explore the potential overlap between the two lists (Rossin et al., 2011).

## Materials and Methods

### Samples

#### OCD Data

The OCD data includes a total of 2998 individuals. This sample consists of 1406 patients that come from 1065 European families. Six-hundred and twenty-one families were recruited at one of the five recruitment sites and the National Institute of Mental Health. Four-hundred and forty-four families were previously evaluated for an earlier study at Johns Hopkins University; one of our collaborating sites. The final sample is made up of 460 complete trios (an affected proband and both parents), 155 pedigrees (a proband and unaffected sibling), and 450 families with a complex family structure. An additional 192 probands that lacked another family member in the study (singletons) were included in the sample as well. The genotyping was performed at the Johns Hopkins SNP Center using Illumina’s HumanOmniExpress bead chips.

#### GENEVA Controls

The Gene Environment Association Studies (GENEVA) seek to identify genetic factors that may play a part in addiction, in the form of a large genome-wide association study. The subjects are DSM-IV alcohol dependent as well as illicit drug dependent cases and controls. The controls are non-dependent and unrelated subjects of European and African American descent. This data is available for download at the dbGaP database (phs000092.v1.p1). One-thousand-two-hundred and ninty-six of the European controls were combined with the OCD data for PRS analyses, as there were very few controls in the OCD data. The samples were genotyped using the Illumina Human 1M platform.

#### ADHD Data

The ADHD dataset was downloaded from the Psychiatric Genomics Consortium (PGC)^2^. This data contains only the summary statistics; no individual level data was included. This data combined four projects: (1) the Children’s Hospital of Philadelphia (CHOP); (2) phase I of the International Multisite ADHD Genetics Project (IMAGE); (3) phase II of IMAGE (IMAGE II); and (4) the Pfizer funded study from the University of California, Los Angeles, Washington University and the Massachusetts General Hospital (PUWMa; Neale et al., 2010a). The total sample consists of 2960 childhood ADHD cases, as well as parental and independent controls.

The CHOP data contains trio families that were recruited from both pediatric and behavioral health clinics in the surrounding Philadelphia area (Elia et al., 2009). Patients were diagnosed based on the K-SADS interview (Kaufman et al., 1997). Trios were included if the families were of European descent and contained a proband with ADHD between the ages of 6 and 18 years.

The IMAGE sample also contained trio families that were collected using a standardized protocol for the collection sites. These sites included countries in Europe: Belgium, Germany, Ireland, Netherlands, Spain, Switzerland, United Kingdom, as well as Israel. At these sites, parents of the affected children were interviewed using the Parent Account of Childhood Symptom (PACS), which is a semi-structured diagnostic interview used to obtain the clinical phenotypes often associated with ADHD (Chen and Taylor, 2006). Additionally, the parents and the teachers completed separate versions of the Conners ADHD rating scales and the Strengths and Difficulties Questionnaire (Goodman, 1997; Conners et al., 1998). Families diagnosed with ADHD were retained for the study sample.

The IMAGE II sample contained some of the samples from the original IMAGE project and combined these with additional samples provided by other sites (Mick et al., 2010). Similar methods to the original IMAGE project were used. The rest of the samples came from several different sites.

One of the sites was located in Germany and thus the families were of German, Caucasian descent. All of the cases met the DSM-IV criteria for an ADHD diagnosis. The affected proband was at least 6 years old, and affected siblings were included if they were at least 6 years of age as well. All of the children were accessed using the Present and Lifetime Version of K-SADS (K-SADS-PL; Kaufman et al., 1997). Parents and teachers also used the DMS-IV based rating scale to confirm the occurrence of symptoms.

Another site in Cardiff contained a sample of children, ages 6–16 years, of British, Caucasian ancestry. Their parents were interviewed using the Parent Child and Adolescent Psychiatric Assessment (CAPA; Angold and Costello, 2000). Additionally, a telephone interview with their teacher was conducted using the Child ADHD Teacher Telephone Interview (CHATTI; Holmes et al., 2003).

The Scottish site included children, ages 6–16 years, of British, Caucasian descent. Patients were accessed through the CAPA as well as the Conners Teacher Rating Scale (Conners et al., 1998; Angold and Costello, 2000).

At the Dutch Site, participants aged 3–18 years were diagnosed with ADHD, oppositional defiant disorder (ODD), conduct disorder (CD), as well as mood and anxiety disorders. A majority of this data was collected for a sibling pair genome-wide linkage study of ADHD. Patients were accessed using the DSM-IV version of the Diagnostic Interview Schedule for Children (DISC-P; Shaffer et al., 2000). The DISC-P was supplemented with Conner’s Questionnaire, the Childhood Behavior Checklist (CBCL), the Teacher Report Form (TRF), and the Strengths and Weaknesses of ADHD Symptoms and Normal Behaviors (SWAN; Geller et al., 1996, 2002; Goodman, 1997; Conners et al., 1998).

The IMAGE II controls consisted of 2653 individuals of European descent. These controls were initially collected for a GWAS of schizophrenia (O’Donovan et al., 2008). The participants were drawn from a US representative survey panel of 60,000 individuals. Participants were screened for psychosis as well as bipolar disorder, but not ADHD.

The PUWMa samples contained information from MGH, Washington University, and UCLA. Three-hundred and nine families were recruited at the MGH clinics. Only the adult subjects who had a lifetime DSM-IV-TR diagnosis of ADHD were enrolled. These enrolled participants were screened using the DSM-IV-TR and Epidemiologic version of the Schedule for Affective Disorders and Schizophrenia (K-SADSE; Ambrosini, 2000).

At the Washington University location, 272 families were selected for a genetic epidemiology study to examine the prevalence as well as heritability of ADHD. The original sample contained 812 male and female twin pairs and six individual twins, aged 7–19 years, identified from the Missouri Family Registry. The families were invited to participate if at least one of the twins experienced at least three inattentive symptoms reported during a screening interview. The Missouri Assessment of Genetics Interview for Children (MAGIC) was used to access both the children and parents (Todd et al., 2003). The DSM-IV ADHD diagnoses were based on parents’ reports about their children.

At the UCLA site, 156 participants were chosen from 540 individuals, aged 5–18 years, and their parents, originally from a sample of 370 families that contained ADHD affected sibling pairs. The children and adolescents were assessed using the K-SADS-PL (Kaufman et al., 1997). The parents were assessed using the Lifetime version of SADS (SADS-LA-IV), as well as the K-SADS Behavioral Disorders module (Kaufman et al., 1997). The Swanson, Nolan, and Pelham, version IV (SNAP-IV) rating scale was used as a direct interview method, as well as the CBCL and TRF (Geller et al., 1996, 2002; Bussing et al., 2008). In addition, parents rated themselves and their spouses’ behaviors using the ADHD Rating Scale IV (Zhang et al., 2005).

### Pre-Imputation Quality Control

Quality control (QC) measures were previously completed in-house for a cross-disorder genome-wide study that used the same OCD dataset. QC was conducted using PLINK^3^ (Purcell et al., 2007). SNPs with <95% call rate or SNPs with <0.01 allele frequency were removed. SNPs with a *p*-value <1 × 10^−6^ for the Hardy Weinberg Equilibrium (HWE) test were removed as well. After QC, 591,322 SNPs remained from the 2998 individuals for statistical analyses. For the GENEVA controls after QC, 849,063 SNPs were left from the 1296 individuals.

### OCD Data Imputation

Imputation was also previously completed for the same cross-disorder genome-wide study. The IMPUTE2^4^ software was used (version 2.1.2) as well as 1000 Genomes (June 2014 Data Release) as a reference dataset (Howie et al., 2009, 2011).The haplotypes were phased using SHAPEIT2^5^ (version v2.r644) to produce the best-guess haplotypes (Delaneau et al., 2012).

### Post Imputation Quality Control (QC)

Post imputation QC was previously completed for a cross-disorder genome-wide study on the OCD dataset. GTOOL^6^ (version v0.7.5) was used to convert the genotype data in the GEN format to the PLINK PED format. The GEN data is listed as a set of three probabilities that correspond to the allele pairs AA, AB and BB. If the largest probability of the three is over the threshold (0.9), then the genotype from the PED file was written as the corresponding allele pair. After QC, the OCD dataset was left with 6,995,151 SNPs from 2998 individuals. The GENEVA controls contained 6,995,151 SNPs from 1296 unrelated individuals.

The ADHD sample was cleaned prior to upload to the site^7^. Therefore, the ADHD sample did not need to undergo additional QC measures.

### Statistical Analysis

Because the OCD dataset has pedigree structure, the initial genome wide association analyses were completed using FBAT^8^ (version v2.0.4) for the OCD dataset (Laird et al., 2000). For the meta-analysis, METAL^9^ was used to conduct the analysis (Willer et al., 2010).

#### Polygenic Risk Score Analyses

PRS analyses were conducted using PRSice^10^ (version v1.23; Euesden et al., 2014). The PRS summarizes the genetic effects of a group of markers (SNPs) that individually do not reach significance in a large association study (Dudbridge, 2013). The risk score was calculated as a sum of single-nucleotide polymorphism alleles that are associated with a specific trait for an individual (Howie et al., 2009). The score was weighted by effect sizes that are estimated from a GWAS. In order to examine the genetic relationship between ADHD and OCD, PRS’s were calculated.

**Figure 1:**
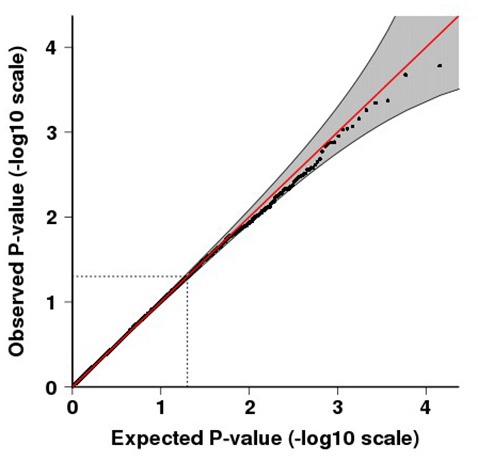
**Quantile-quantile (QQ) plot for *p*-values of the meta-analysis.** QQ plots compare the observed vs. expected test-statistic distributions. The shading indicates the 95% confidence intervals. The inflation factor, *λ* is 1.008.

For this study, ADHD was used as the discovery sample because that sample size was larger and only the summary statistics were available for this analysis. Using OCD as the target dataset and ADHD as the discovery sample, the PRSice software was used to calculate these scores. PRSice was designed to automate the steps of the PRS analyses by using both PLINK (Purcell et al., 2007) and R (R Core Team, 2015). Linkage disequilibrium (LD) pruning was completed, using *p*-value thresholds of *p* < 0.01, 0.1, 0.2, 0.3, 0.4 and 0.5. PRSice also automates the LD pruning process. Within each of these LD thresholds, *p*-value significance thresholds were determined, and *R*^2^ values were calculated based on how well the regression fits the data, which can be seen in Figure 1. For each of these *p-value* significance thresholds quantitative polygenic scores were calculated for each individual within the target data. These scores were calculated by multiplying the number of risk alleles for each SNP (0, 1, or 2) by the score for that same SNP, estimated from the discovery sample.

#### Protein-Protein Link Evaluation

DAPPLE was used to conduct a protein-protein analysis to examine the connectivity between potential associated proteins (Rossin et al., 2011). DAPPLE seeks to find significant physical connectivity between proteins that are encoded by the genes found in the loci associated with the disease. These protein-protein interactions are based on reported biological information between proteins in InWeb, a database of 169,810 high-confidence pairwise interactions involving 12,793 proteins (Rossin et al., 2011). To test for the non-randomness of these protein connections, DAPPLE was used to create random protein interaction networks with a within-degree node-label permutation method. Random networks hold the same size, number of edges and number of proteins with the same number of connections as the original network. Protein names in the random networks, however, are randomly reassigned to proteins of equal protein connectivity, allowing for the evaluation of non-randomness in the original network based on protein binding degree (Rossin et al., 2011).

#### Expression Quantitative Trait Locus (eQTL) Analysis

A *p*-value threshold of *p* < 1.00 × 10^−4^ was used in order to examine the relationship between the candidate SNPs and relevant eQTLs, using the eEQLAnalysis^11^ software. eEQLAnalysis can be used to conduct an eQTL analysis for the selected SNP list based on the Brain Cloud^12^ data set (GSE30272). This dataset contains both SNP data and gene expression data from 268 healthy subjects. The software includes three modules: eQTL Map Generation; Permutation for Selected eQTLs; and report generation. The input of the software provides a list of nominated SNPs. The outputs include the “significant SNPs” and associated statistics including eQTL *p*-values and permutation based *p*-values. For more information about the software, please refer to: http://hongbaocao.gousinfo.com/Software4Download.html. The eQTL analysis we conducted seeks to link SNPs to regions of the prefrontal cortex.

## Results

### Meta-Analysis

**Figure 2:**
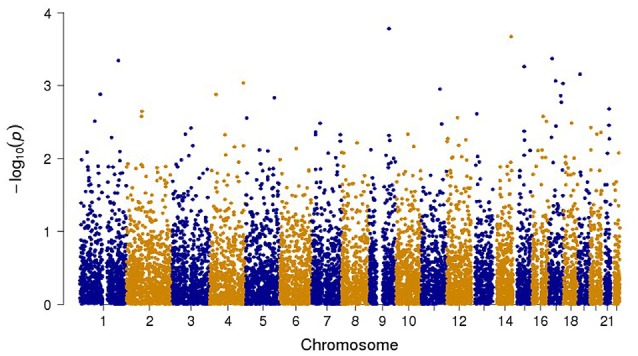
**Manhattan plot of all the genotyped and imputed SNPs for *p*-values of the meta-analysis between the attention deficit hyperactivity disorder (ADHD) and obsessive compulsive disorder (OCD) studies**.

The final combined dataset consisted of 2998 OCD samples and 5415 ADHD samples. A total of 6,598,140 SNPs were analyzed in the meta-analysis. Both the quantile-quantile (QQ) plot and Manhattan plot show the association *p*-values from the meta-analysis (Figures 1, 2, respectively). The QQ plot estimates if two datasets come from populations that share a common distribution (Figure 1). It compares the observed vs. expected distributions of the test statistics. The genomic control inflation factor, *λ* in this analysis is 1.008, which provides no evidence for residual population stratification. The corresponding *p*-values from the meta-analysis of both the genotyped and imputed SNPs are shown in the Manhattan plot (Figure 2). None of the SNPs reached genome wide significance. A list of the top SNPs can be found in Table 1. The most significant SNP was rs10989904 with a *p*-value of 1.65 × 10^−4^. This SNP occurs in an intergenic region.

**Table 1:** **Top SNPs from the meta-analysis**.

Chr	SNP	Position	A1/A2	Gene name	*P*-value	Direction
9	rs10989904	104030215	T/G		1.65E-04	−−
14	rs234601	96146269	T/C		2.12E-04	−−
17	rs11656215	17393792	T/C	PEMT	4.25E-04	−−
1	rs708723	204005889	T/C	RAB29, NUCKS1, SLC41A1, SLC45A3	4.52E-04	++
15	rs7167122	58162157	T/C		5.48E-04	−−
19	rs375323	11247225	T/C	DOCK6	6.94E-04	−−
17	rs1901187	35899673	T/C	TNS4	8.59E-04	++
4	rs4557213	177926127	A/G	VEGF-C	9.18E-04	++
17	rs4073996	75056346	T/C		9.33E-04	−−
11	rs2656198	98868897	A/G		1.11E-03	−−

*Chr, chromosome; SNP, single nucleotide polymorphism; Position, chromosome positions at hg19, A1, reference allele, A2, alternative allele, GeneName, genes including SNPs, P-value, meta-analysis p-value, Direction, summary of the effect of the direction for each study*.

### Polygenic Risk Score Analyses

**Figure 3:**
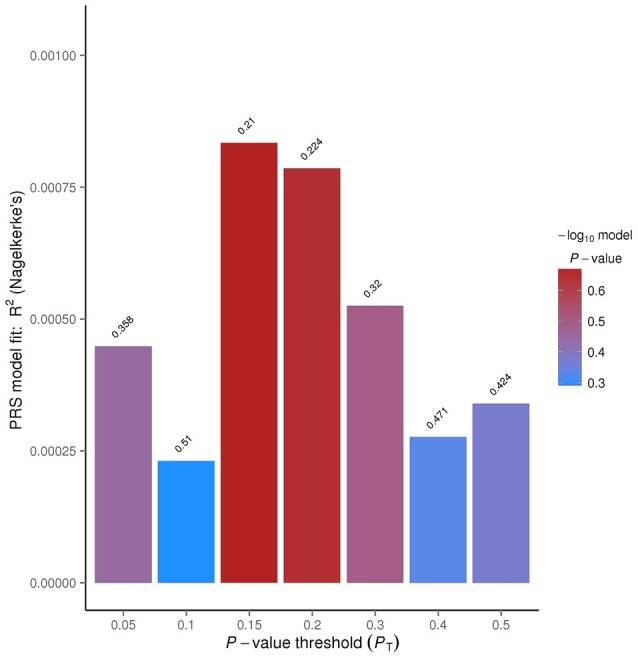
**PRSice bar plot for linkage disequilibrium (LD) threshold of 0.1.** The tallest bar indicates the best fit polygenic risk score (PRS) for the ADHD PRS predicting OCD.

The polygenic risk model was tested on the target sample to obtain the PRS for each individual. Logistic regression was conducted to examine the relationship between risk score and the case-control status of the target data. The percentage of the phenotypic variance that can be explained by the risk score was automated by PRSice (Figure 3).

The *R*^2^ value indicates how well the logistic regression approximates the data, based on the *p*-value thresholds. The *p*-value threshold of 0.15 had an *R*^2^ value of 0.0834%. This means that approximately 0.08% of the data overlapped between the OCD and ADHD data.

### Protein-Protein Link Analysis

**Figure 4:**
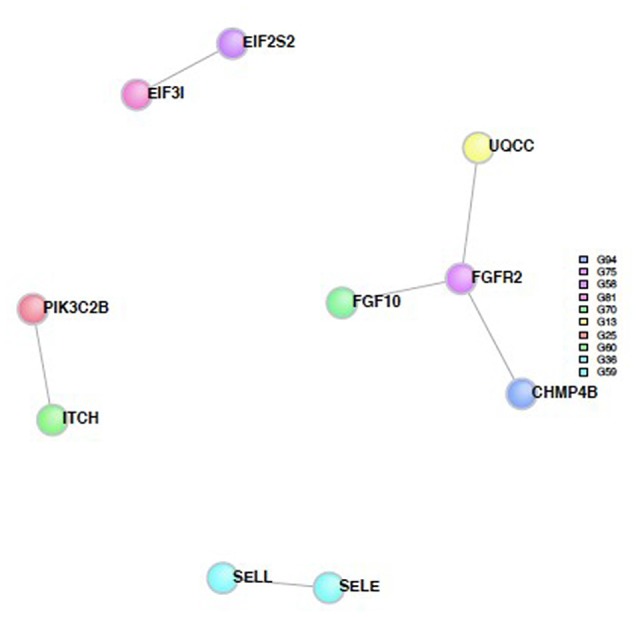
**Protein-Protein interaction network built from proteins from the SNPs from the meta-analysis.** The colored circles represent the proteins and the different colors are associated with different regions. The gray lines represent the direct connections between the proteins.

SNPs with a *p*-value <0.001 were included in the analysis to investigate whether any protein(s) associated with the disorders would give a statistically significant *p*-value; A total of 123 genes were included in the analysis based on this criterion. Six direct protein-protein interactions were identified and included ten proteins in all (*CHMP4B, EIF2S2, EIF3I, FGF10, FGFR2, ITCH, PIK3C2B, SELE, SELL and UQCC*; Figure 4). The direct connections are also shown in Table 2. The overall direct connections protein interaction network had a *p*-value of 0.0879 (Figure 4). Additionally, 543 indirect connections contributed to the network that linked the six direct protein interactions. None of the indirect connectors were of known biological relevance based on our current understanding of the diseases. A similar DAPPPLE analysis was conducted previously on an ADHD sample that found no direct connections (Zayats et al., 2015).

**Table 2:** **Results of the protein-protein link evaluation in Disease Association Protein-Protein Link Evaluator (DAPPLE), direct interactions**.

Protein	Region	Uncorrected *p*-value	Corrected *p*-value	Binding protein/s	Function
FGF10	G70	0.00199	0.00199	FGFR2	Fibroblast growth factor 10
				FGF10, UQCC,	
FGFR2	G75	0.01394	0.01394	CHMP4B	Fibroblast growth factor receptor 2 Ubiquinol-Cytochrome C reductase complex
UQCC	G13	0.08598	0.08598	FGFR2	Assembly factor 1
EIF3I	G81	0.11629	0.11629	EIF2S2	Eukaryotic translation initiation factor 3
SELE	G59	0.11629	0.11629	SELL	Selectin E
EIF2S2	G58	0.16079	0.16079	EIF3I	Eukaryotic translation initiation factor 2
SELL	G36	0.15529	0.15529	SELE	Selectin L
CHMP4B	G94	0.18442	0.18442	FGFR2	Charged multivesicular body protein 4A
PIK3C2B	G25	0.16444	0.16444	ITCH	Phosphatidylinositol-4-phosphate 3-kinase
ITCH	G60	0.24461	0.24461	PIK3C2B	Itchy E3 ubiquitin protein ligase

*The p-values are the probability that by chance the individual interactions would be connected to the seed proteins as observed in the network (Figure 4)*.

### Exploring Polygenic Risk for ADHD and OCD

A cross-disorder PRS analysis was completed using the ADHD data as the discovery sample and the OCD data and GENEVA controls as the target sample. An LD pruning threshold was set at 0.1. The predetermined significance thresholds were *p* < 0.05, 0.1, 0.15, 0.2, 0.3, 0.4 and 0.5. The corresponding numbers of SNPs for each threshold were as follows: 11,867 (4.3%), 20,929 (7.7%), 28,664 (10.5%), 35,776 (13.1%), 48,191 (17.7%), 59,096 (21.7%) and 68,295 (25.0%). The 20,929 SNPs for *p* < 0.1 in the discovery sample, contributed 0.023% variance in the target sample. The low variance could be related to the small number of SNPs included in the analysis. 28,664 SNPs for *p* < 0.15 in the discovery sample, contributed 0.083% of the variance explained by OCD. The other groups of SNPs contributed between 0.028%–0.079% of the variance explained by OCD.

### Expression Quantitative Trait Locus (eQTL) Analysis

SNPs with a *p*-value <1.00 × 10^−4^ from the genome wide association tests were included in an eQTL analysis. An eQTL analysis was conducted in order to compare the results with the proteins identified from DAPPLE. The top SNPs associated with the prefrontal cortex are listed in Table 3.

**Table 3:** **Results of the expression quantitative trait locus (eQTL) analysis in eEQLAnalysis, top genes**.

Gene name	Chromosome number	eQTL *p*-value	Permutation *p*-value
LINC00314	21	1.481E-08	0.0039
CXCR2	2	5.934E-08	0.0029
ASB17	1	6.149E-07	0.0088
SELE	1	6.581E-07	0.0000
ACOT7	1	8.591E-07	0.0003
PRPS1L1	7	1.324E-06	0.0004
ZBF580	19	1.431E-06	0.0027
TAS2R41	7	1.663E-06	0.0049
ADAMTS20	12	1.710E-06	0.0027
WDFY3	4	2.136E-06	0.0041

## Discussion

Psychiatric disorders, such as ADHD and OCD, are extremely complex and clinically heterogeneous. However, it has been reported that ADHD and OCD may share common sub-phenotypes. For example, Palumbo et al. (1997) suggested that ADHD, OCD and autism have overlapping etiologies, and thus are interrelated. Additionally, Anholt et al. (2010) found that inattention plays a key role in obsessive-compulsive symptoms and may further link ADHD and OCD. Despite the clinical overlap between ADHD and OCD, the genetic overlap found in this study was limited. It is possible that the heterogeneity of each of the samples diluted the association signals in the meta-analysis, and masked the genetic overlap.

To summarize our findings, we conducted a meta-analysis between ADHD and OCD. The SNP rs10989904 had the strongest association signal (*p*-value = 1.65 × 10^−4^). This SNP is in an intergenic region, but is near the LOC100127962 pseudo gene. None of the other SNPs were in any known biologically relevant genes. The GWAS conducted on the ADHD PGC data found the most significant SNP on the CDH13 gene (Neale et al., 2010b). This gene was not identified in the meta-analysis. We then used DAPPLE to identify any network of proteins associated with the two disorders. This analysis found that *CHMP4B, EIF2S2, EIF3I, FGF10, FGFR2, ITCH, PIK3C2B, SELE, SELL and UQCC* all contributed to disease susceptibility. *EIF2S2* and* FGFR2* were found to be associated with both disorders. Zayats et al. (2015) reported that *EIF2S2* may participate in a protein network that is impaired in individuals with ADHD.

Another protein found in the direct network, *FGFR2*, was reported to be associated with hyperactive behavior in mice deficient in the protein (Kaga et al., 2006). Schubert et al. (2014) also found that *FGFR2* is involved in generating the excitatory glutamatergic pyramidal neurons in the medial prefrontal cortex. The prefrontal cortex has been implicated in the pathophysiology of several neurodevelopmental disorders, including ADHD and OCD, and both disorders exhibit impairments in inhibitory control (Schubert et al., 2014). However, Norman et al. (2016) found that the two disorders exhibited different underpinnings for this impairment.

The eQTL results were compared to the findings from the DAPPLE analysis. Only one gene overlapped between DAPPLE and eQLAnalysis. The overlapping gene was *SELE*, which plays a role in inflammation. Inflammation, especially in the brain, has been found to be associated with the development of neuropsychiatric disorders. Specifically, in populations of youth and adolescents, there were elevated markers for inflammation among several neuropsychiatric disorders including ADHD and OCD (Mitchell and Goldstein, 2014).

More interestingly, the WDFY3 gene was found to be associated with axon guidance in mice, which was previously reported to be associated with ADHD (Dragich et al., 2016). A phenotype of ADHD was shown to be correlated with a failure in axon guidance (Mooney et al., 2016). The WDFY3 gene had an eQTL *p*-value of 2.136 × 10^−6^ and a permuted *p*-value of 0.0041 (Table 3).

Our study also showed that SNPs with a *p*-value <0.15 from the discovery sample contributed 0.083% of the variance explained by OCD. In general, psychiatric disorders such as OCD and ADHD are likely to arise from the influence of a large number of susceptibility genes across the genome, as well as the proportion of OCD phenotypic variance explained directly by the targeted SNP’s (Yu et al., 2015).

Notably, our study has a limited sample size for both the ADHD and OCD samples, and thus are likely to be underpowered in detecting statistically robust polygenic score effects and signals in the meta-analysis. In the future, a larger sample size may provide a more accurate PRS and better estimate of heritability. Additionally, the samples may exhibit heterogeneity that each of these diseases are clinically heterogeneous.

Clinical heterogeneity tends to increase as the number of data collection sites increases. A study by Anttila et al. (2016) examined the genetic correlation between neurological and psychiatric disorders including ADHD and OCD. It should be noted that the ADHD dataset used in our analysis was a subset of the data used in the Anttila et al. (2016) analysis. Our results were consistent with that of Anttila et al. (2016); no significant genetic correlation was found between the two disorders.

Although the analyses conducted including meta, PRSs, DAPPLE provide little evidence to suggest that ADHD and OCD share common genetic etiologies, our eQTL analysis suggested a potential role for the WDFY3 gene in psychiatric disorders such as ADHD and/or OCD. We also anticipate that more genes/pathways will emerge with future studies of larger sample sizes.

## Author Contributions

MLR, WG, GN and YYS co-designed and completed the statistical analysis. MLR wrote the manuscript. MLR, PSN, GN and YYS edited the manuscript. JFS, YW, PSN, JK, MAG, MAR, BC, JB, FSG, AEP, BDG, AJF, JTM, DAG, DLM, JAK, SAR, NCM, ELN, KDA, JP, DLP, ES, BM, MM, JQ and GN collected data, provided information for the OCD dataset, attended many phone discussions and contributed to the writing and data interpretation.

## Conflict of Interest Statement

The authors declare that the research was conducted in the absence of any commercial or financial relationships that could be construed as a potential conflict of interest. The reviewer YSJ and handling Editor declared their shared affiliation, and the handling Editor states that the process nevertheless met the standards of a fair and objective review.

## Footnotes

^1^http://www.broadinstitute.org/mpg/dapple/dapple.php

^2^http://www.med.unc.edu/pgc/downloads

^3^http://zzz.bwh.harvard.edu//plink/

^4^https://mathgen.stats.ox.ac.uk/impute/impute_v2.html

^5^https://mathgen.stats.ox.ac.uk/genetics_software/shapeit/shapeit.html

^6^http://www.well.ox.ac.uk/~cfreeman/software/gwas/gtool.html

^7^https://www.med.unc.edu/pgc/results-and-downloads

^8^http://www.hsph.harvard.edu/fbat/fbat.htm

^9^http://csg.sph.umich.edu/abecasis/Metal/

^10^http://prsice.info/

^11^http://hongbaocao.gousinfo.com/Software4Download.html

^12^http://braincloud.jhmi.edu/
